# Congenital anomalies during the 2015–2018 Zika virus epidemic: a population-based cross-sectional study

**DOI:** 10.1186/s12889-022-14490-1

**Published:** 2022-11-12

**Authors:** Fabio Antonio Venancio, Maria Eulina Quilião, Danielli de Almeida Moura, Micael Viana de Azevedo, Sahra de Almeida Metzker, Lisany Krug Mareto, Márcio José de Medeiros, Cláudia Du Bocage Santos-Pinto, Everton Falcão de Oliveira

**Affiliations:** 1 Programa de Pós-Graduação em Doenças Infecciosas e Parasitárias, Universidade Federal de Mato Grosso do Sul, Campo Grande, MS Brasil; 2 Centro Especializado em Reabilitação da Associação de Pais e Amigos dos Excepcionais, Campo Grande, MS Brasil; 3 Faculdade de Medicina, Universidade Federal de Mato Grosso do Sul, Campo Grande, MS Brasil; 4 Instituto Integrado de Saúde, Universidade Federal de Mato Grosso do Sul, Campo Grande, MS Brasil; 5 Universidade Federal do Rio de Janeiro, Campus Macaé, Rio de Janeiro, RJ Brasil

**Keywords:** Birth defects, Congenital anomalies, Epidemic, Microcephaly, Zika virus

## Abstract

**Background:**

Congenital anomalies are associated with several clinical and epidemiological complications. Following the Zika epidemic onset in Latin America, the incidence of congenital anomalies increased in Brazil. This study aimed to determine the frequency of congenital anomalies in one Brazilian state and assess potential factors associated with them.

**Methods:**

This cross-sectional descriptive study was based on data concerning congenital anomalies recorded in the Brazilian Live-Born Information System during the Zika epidemic in Mato Grosso do Sul state from 2015 to 2018. Congenital anomalies were stratified according to year of birth and classified using ICD-10 categories.

**Results:**

In total, 1,473 (0.85%) anomalies were registered. Within the number of cases recorded, microcephaly showed the greatest frequency and variations, with a 420% increase observed in the number of cases from 2015 to 2016. We identified an increase in the incidence of central nervous system anomalies, with the highest peak observed in 2016 followed by a subsequent decrease. Musculoskeletal, nervous, and cardiovascular system anomalies, and eye, ear, face, and neck anomalies represented 73.9% of all recorded anomalies. There was an increased chance of congenital anomalies in uneducated (odds ratio [OR] 5.56, 95% confidence interval [CI] 2.61–11.84) and Indigenous (OR 1.32, 95% CI 1.03–1.69) women, as well as among premature births (OR 2.74, 95% CI 2.39–3.13).

**Conclusions:**

We estimated the incidence of congenital anomalies during the Zika epidemic. Our findings could help to support future research and intervention strategies in health facilities to better identify and assist children born with congenital anomalies.

**Supplementary Information:**

The online version contains supplementary material available at 10.1186/s12889-022-14490-1.

## Background

Congenital anomalies are a set of functional and morphological abnormalities occurring during embryonic development due to several etiologies [1–4]. Approximately 65% of congenital anomalies have unknown causes, 25% have genetic causes, and 10% have environmental and maternal causes [1, 2]. These occurrences represent major concerns due to their effects on maternal and child health [5].

In the 1960s, more than 10,000 cases of serious congenital malformations were recorded in live births due to the use of thalidomide by pregnant women [6–8]. Since then, several countries have actively invested in surveillance programs and systems to detect congenital anomalies [9, 10].

It is estimated that 7.9 million live births worldwide have some type of serious congenital anomaly annually and that 295,000 children die within the first 28 days due to congenital anomalies [3, 10]. Congenital anomalies account for a staggering 25.3–38.8 million disability-adjusted life years worldwide [11, 12] due to the high incidences and severity of the numerous anomalies. However, in relation to these anomalies, there has been a lack of public policy formulations, a limited number of relevant and prioritized social policies, limited specialized assistance for individuals and families, and low incentives in the area of scientific research [13, 14] In Brazil, 130,636 live births with congenital anomalies were registered from 2015 to 2018 in the Brazilian Live-Born Information System (*Sistema de Informações sobre Nascidos Vivos*, SINASC) during the immediate postpartum period [15].

Among the various possible causes of congenital anomalies, infectious agents appear to be the most important [12]. The pathogens most frequently associated with intrauterine infections are the Zika virus (Z); the bacterium *Treponema pallidum,* which causes syphilis (S); the protozoan *Toxoplasma gondii,* which causes toxoplasmosis (TO); the rubella virus (R); the cytomegalovirus (C); and the herpes simplex virus (H), constituting the acronym Z-STORCH [16, 17].

Several clinical and epidemiological complications are associated with congenital anomalies. ZIKV infections during pregnancy resulted in an increase in the incidence of congenital anomalies between 2015 and 2018 in Brazil [18]. Moreover, globally, it is estimated that gestational syphilis is associated with adverse outcomes in more than one million pregnancies, with approximately 300,000 fetal and neonatal deaths per year [3]. Considering the imminent risk of the development of new cases with structural and neurodevelopmental birth defects, especially as a result of prenatal care disruptions observed during the COVID-19 pandemic [1, 19, 20] and the risk of the development of new Zika virus epidemics [21–23], it is essential to identify the clinical and epidemiological profiles of these cases. Therefore, this study aimed to describe the frequency of congenital anomalies in the Brazilian state of Mato Grosso do Sul and analyze factors potentially associated with congenital anomalies.

## Methods

This was a cross-sectional descriptive study based on data relating to congenital anomalies obtained from the SINASC. These data refer to congenital anomalies diagnosed at birth in Mato Grosso do Sul from 2015 to 2018. In the Brazilian surveillance system, congenital anomalies identified in the clinical examination of newborns are recorded after each live birth [24]. The form used for recording, namely, the Declaration of Live Birth, is a tool intended to certify the birth of a newborn and provide information on the characteristics of the birth, which is then used to establish specific health indicators.^25^ The information recorded is then entered into the SINASC. Since 1990, the SINASC has become the official system for registering congenital anomalies in Brazil [25].

The Research Ethics Committee of the Federal University of Mato Grosso do Sul approved this study (CAAE: 91,326,518.10000.0021; Registration No.: 3.298.330).

Data were collected and analyzed concerning congenital anomalies in live-born babies registered in the SINASC and identified using ICD-10 categories (Supplementary Table 1). Congenital anomalies were classified into 11 categories according to chapter XVII of the ICD-10 [26], namely, anomalies of the central nervous system, head and neck anomalies, anomalies of the cardiovascular system, anomalies of the respiratory system, oral clefts, digestive system anomalies, abnormalities of the genital system, urinary system anomalies, musculoskeletal anomalies, chromosome disorders, and anomalies not classified in other systems.

The following maternal, gestational, and birth data were analyzed: maternal age (up to 19 years, 20–34 years, 35–39, or ≥ 40 years) [27, 28], prenatal consultations (yes or no), ethnicity (European, non-European, or Indigenous), education (yes or no), type of pregnancy (single, multiple), duration of pregnancy in weeks (pre-term, term, or post-term) [29], sex of the child (male or female), Apgar score at the first and fifth minute (0–2, 3–7, or 8–10 points), and birth weight (low weight, normal, or macrosomic). Cases of incompletely recorded anomalies that could not be classified based on ICD-10 categories were excluded. Relevant missing or indeterminate data were also excluded from the association analysis.

A descriptive analysis was used to characterize cases with congenital anomalies that had been registered in the SINASC, which were then classified and grouped according to ICD-10 categories. The incidences were expressed as per 10,000 births for each category [26, 30] and stratified according to year of birth. To estimate the incidence of congenital anomalies at birth, the ratio between the total number of live-born babies with congenital anomalies included in the registration and the total number of births recorded in the SINASC was calculated.

Statistical analysis was performed using R (version 4.1.1) using the RStudio interface [31]. Associations were evaluated using odds ratios (OR) with 95% confidence intervals (CIs) and chi-square tests set at a significance level of 0.05. Two groups were compared for the association analysis, namely, one group of live births registered with congenital anomalies and one group of live births with no record of congenital anomalies. The independent variables used were maternal and gestational characteristics (age, ethnicity, education, prenatal care, type of pregnancy, and duration of pregnancy) and certain characteristics related to the child (Apgar score and birth weight). Congenital anomalies were the dependent variable. The R package ggplot2 was used to plot graphs [32], and the R package epitools was used to perform the association analysis [33]. The study results are reported according to STROBE guidelines.

## Results

A total of 172,960 births were registered in the SINASC in Mato Grosso do Sul from 2015 to 2018. Of these, 1,116 (0.64%) newborns had one or more types of congenital anomalies. Among the types of anomalies, 1,473 were registered and divided into 74 classifications according to ICD-10 categories.

In terms of congenital anomalies with external structural defects identifiable on clinical examination, congenital deformities of the feet were most common (Q66, 12.4%), followed by polydactyly (Q69.9, 8.1%). Congenital anomalies associated with greater severity were less frequent, such as gastroschisis (Q79.3, 4.4%), microcephaly (Q02.X, 2.8%), hydrocephalus (Q03, 2.7%), anencephaly (Q00.0, 2.5%), heart disease (Q24, 2.5%), and Down syndrome (Q90.9, 2.2%). Microcephaly showed the greatest variations in terms of the number of cases recorded each year. There were five cases in 2015, 26 cases in 2016, eight cases in 2017, and only three cases in 2018, indicating a 420% increase in the number of cases from 2015 to 2016 (Supplementary Table 1).

**Fig. 1:**
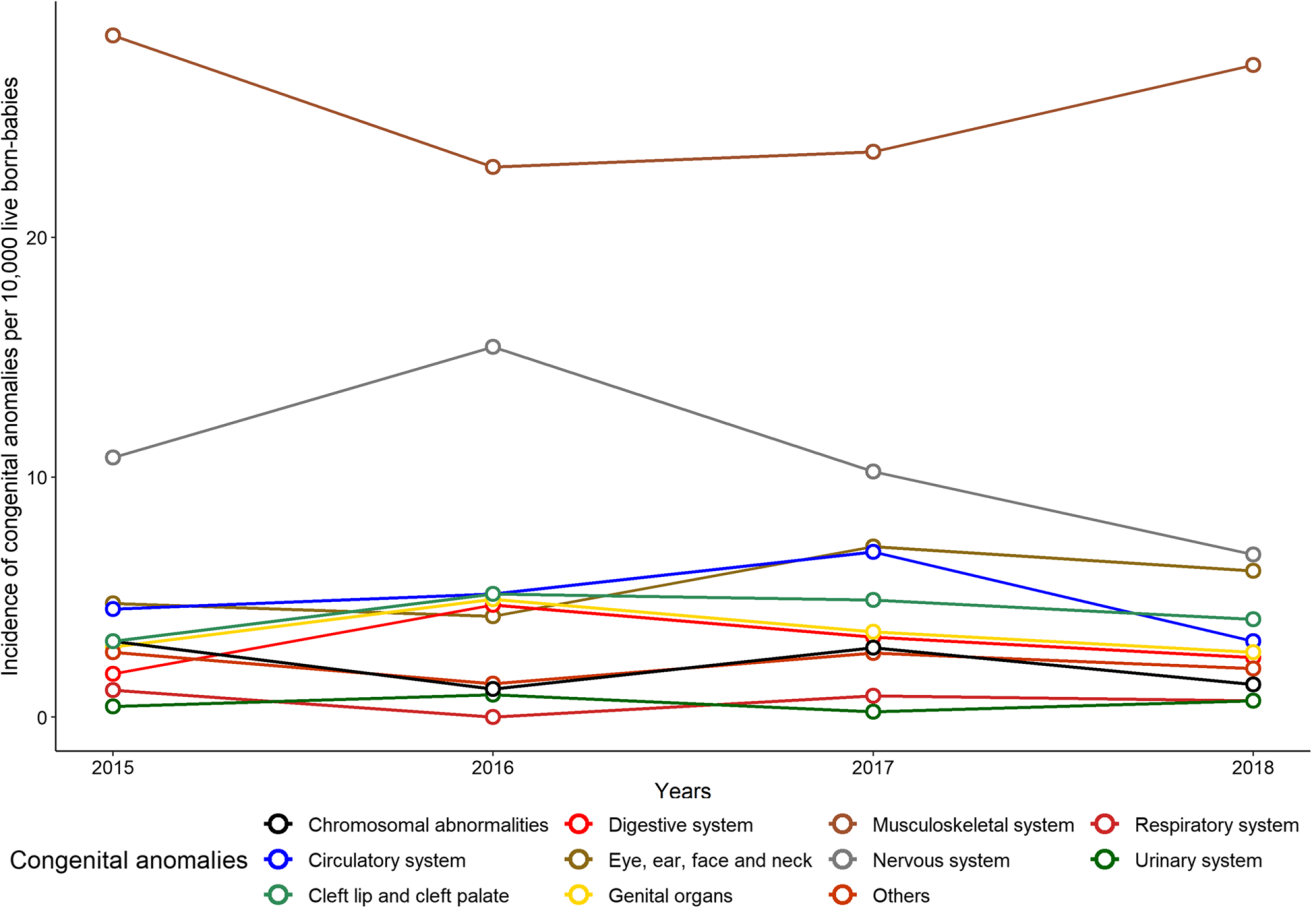
Incidence of congenital anomalies per 10,000 live-born babies

Among the four periods evaluated, we observed an increase in the incidence of central nervous system anomalies in 2016 (incidence rate, 15.4 per 10,000 births) compared with 2015 (incidence rate, 10.8 per 10,000 births), 2017 (incidence rate, 10.2 per 10,000 births), and 2018 (incidence rate, 6.7 per 10,000 births) (Fig. 1 and Supplementary Table 2). Birth defects of the musculoskeletal system had the highest incidence in the four years evaluated; however, they decreased during the ZIKV epidemic period in 2016 (incidence of 22.9 per 10,000 births), followed by an increase in the subsequent years. Anomalies of the musculoskeletal, nervous, and cardiovascular systems and eye, ear, face, and neck anomalies represented 73.9% of all anomalies recorded during this period.

Supplementary Table 3 summarizes the frequencies of congenital anomalies distributed according to ICD-10 categories, in addition to maternal, gestational, and birth data. Among the cases with reported chromosomal abnormalities, 44.1% were reported in live-born babies to women aged ≥ 35 years. Of nervous system anomalies, 61.7% and 11.5% were registered in live-born babies to non-European and Indigenous mothers, respectively. Of children with central nervous system anomalies, 3.5% were born to women who had no prenatal care. Among genital anomalies, 82.7% were identified in male children. In terms of Apgar scores, 40.7% of live-born babies with respiratory system anomalies and 15.9% of those with central nervous system anomalies had Apgar scores that ranged from 0 to 2 in the first minute of life.

When analyzing factors that may be associated with congenital anomalies, we identified an increased likelihood of congenital anomalies in uneducated women (OR 5.56, 95% CI 2.61–11.84), Indigenous people (OR 1.32, 95% CI 1.03–1.69), women who did not receive prenatal care (OR 1.76, 95% CI 1.20–2.58), women with a multiple pregnancy (OR 1.80, 95% CI 1, 30–2.48), and women with premature births (OR 2.74, 95% CI 2.39–3.13) (Table 1).

**Table 1:** Maternal, gestational, and birth data according to the occurrences of congenital anomalies in live-born babies. Mato Grosso do Sul, Brazil (2015–2018)

	Congenital anomalies		Odds ratio (95% CI)^a^
	Yes	No	
**Age (mother)**			
up to 19 years	212	31,579	1.07 (0.92, 1.25)
20–34 years	752	119,788	Reference
35–39 years	122	16,584	1.17 (0.97, 1.42)
≥ 40 years	30	3702	1.29 (0.89, 1.86)
**Ethnicity**			
European	367	61,406	0.89 (0.78, 1.01)
Non-European	678	101,042	Reference
Indigenous	71	8015	1.32 (1.03, 1.69)
**Prenatal care**			
No	27	2386	1.76 (1.20, 2.58)
Yes	1089	168,907	Reference
**Schooling**			
No	7	197	5.56 (2.61, 11.84)
Yes	1109	166,902	Reference
**Pregnancy (type)**			
Single	1.077	168,232	Reference
Multiple	39	3393	1.80 (1.30, 2.48)
**Gestational age (weeks)**			
< 37 weeks	294	19,867	2.74 (2.39, 3.13)
37–41 weeks	791	146,266	Reference
≥ 42	31	4496	1.27 (0.89, 1.83)

*CI* Confidence interval

^a^Pearson’s chi-squared test

## Discussion

Our data describe cases of congenital birth anomalies reported in Mato Grosso do Sul during the Zika epidemic in Brazil between 2015 and 2018, and we identified 74 types of congenital anomalies recorded at birth and high frequencies of external and internal structural defects (Supplementary Table 1). Approximately 66% of the congenital anomalies worldwide have unknown causes [2, 33]. Official data from the Brazilian Ministry of Health show that approximately 24,000 newborns with birth defects are reported annually to the SINASC; however, this number is likely to be an underestimation due to the absence of a national investigation system for these events [34].

The sudden increase in the incidence of congenital anomalies of the central nervous system in 2016 followed the increase in cases of ZIKV fever and congenital Zika syndrome (CZS) at our study location [35]. This trend was also reflected in several other regions of Brazil, where an increase in congenital abnormalities was reported, which then subsequently declined [36]. Anomalies of the musculoskeletal system were reported most frequently, which was consistent with findings reported nationally [36]. We cannot explain why the incidence in musculoskeletal deformities decreased in 2016. Similar to arthrogryposis being a clinical finding in CZS [37], we assumed that some cases with musculoskeletal deformities were no longer reported in the SINASC and only reported in the Registros de Eventos em Saúde Pública (RESP-Microcephaly), referred to as RESP henceforth) for the investigation of CZS during this period. This hypothesis is supported by Venancio et al.’s findings [35]. They investigated and ruled out approximately 70% of the suspected cases of CZS reported in the RESP in the state.

The incidence of general anomalies in 2016 in this study was 66.29 per 10,000 births, which was lower than that reported in Brazilian Ministry of Health national data for the same period (115 per 10,000 births), as well as in published data for Brazilian states such as in Sergipe and Pernambuco (incidence of 149–169 per 10,000 births) [36]. The higher incidence of ZIKV-related microcephaly in these states possibly influenced these findings [36].

When comparing the frequency of congenital anomalies in Brazil with data from other international surveys, we observed that certain states within the United States, such as Florida (prevalence of 248.7 per 10,000 births) and Texas (prevalence of 465.7 per 10,000 births), recorded higher incidences of congenital anomalies [38, 39]. This was also observed in European and Japanese regions and may be associated with superior medical infrastructure allowing better tracking of congenital anomalies [40, 41]. When comparing the incidences of congenital anomalies with less developed regions, we also observed higher prevalence rates in some localities, such as Entebbe in Uganda (761 per 10,000 births) [42], and Ogbomoso in Nigeria (630 per 10,000 births) [43]. These countries are in regions with a high prevalence of sexually transmitted diseases such as syphilis, which might have contributed to the increased number of cases [44].

Brazil was the epicenter of a global public health crisis from 2015 to 2018, with an increase in cases of ZIKV-related microcephaly in newborns [45]. Approximately 18,578 children were monitored, and 3,496 cases of CZS were confirmed in the children born during this period [46]. The main infection-associated congenital anomalies are known to be directly linked to damage to the nervous [47, 48], auditory, ocular [49], musculoskeletal [47], and cardiac [48] systems. They are usually related to *Toxoplasma gondii* [47], *Treponema pallidum* [48], varicella-zoster virus [48], rubella virus [48, 50], cytomegalovirus [48, 51], herpes simplex virus [48, 52], and ZIKV [17, 30] infections.

Z-STORCH infections constitute a group of perinatal infections that may have similar clinical features. They are a significant cause of fetal and neonatal mortality and a major cause of infant morbidity [53]. The prevalence of congenital anomalies related to Z-STORCH infections have been reported to be as follows: syphilis, 7.8 per 10,000 births; toxoplasmosis, 1.0 per 10,000 births [53]; rubella, 0.1 per 10,000 births [54]; cytomegalovirus, 1.4 per 10,000 births [55]; and ZIKV, 3.8 per 10,000 births in the northeast and 2.0 per 10,000 births in the center-west regions of Brazil [35, 56].

Among the types of congenital anomalies described in this study, there may be similarities in clinical findings in children exposed to any of the Z-STORCH pathogens. For example, some infants with severe microcephaly following exposure to cytomegalovirus have been characterized as having a marked reduction in the height of the cranial vault with overlapping sutures and a redundant scalp with wrinkles or folds [30, 57]. This outcome is indistinguishable from that of CZS by physical examination [30]. It is estimated that 4–10% of congenital anomalies are related to environmental and maternal factors, including infectious agents [2]. From 2015 to 2018, 1,485 cases of congenital syphilis were recorded in Mato Grosso do Sul [58], and Brazil experienced a shortage of penicillin [59, 60], an antibiotic used in the treatment of syphilis. In congenital syphilis, some of the anomalies observed at birth are also observed in CZS, such as hydrocephaly and congenital malformations of the eye, ear, and brain [17, 30].

Our findings showed an increase in the number of registered cases of congenital anomalies of the central nervous system in Mato Grosso do Sul in 2016 (incidence of 17.3 per 10,000 births), well below the prevalence recorded in the northeast region (23.9 per 10,000 births), which reported the highest incidences of microcephaly in the country during the ZIKV epidemic [46, 61]. However, the incidence remained above the national average (10.6 per 10,000 births) [62]. The increased number of congenital nervous system anomalies in this period was driven by a 420% growth in the number of microcephaly cases from 2015 to 2016, which can be attributed to the recorded circulation of ZIKV in the center-west region that consequently led to the highest incidence of ZIKV fever in the general population in Brazil [36].

Through restricting the comparison of our findings to the group with the most common congenital anomalies found after maternal exposure to ZIKV, such as nervous system, eye, and ear anomalies [63], we observed that some states in the United States, such as Texas and Massachusetts, had a higher prevalence of anomalies of nervous system (20.5–36.7 per 10,000), eye, and ear anomalies (8.03–18.9 per 10,000) [38, 39], which was higher than those identified in this study even during the ZIKV epidemic in Brazil. This may be related to the surveillance model adopted in the United States, which involves reviewing medical records in maternity hospitals and in pediatric units, routine follow-ups of all newborns aged > 20 weeks in intensive care units, and cross-matching data between genetic laboratories [45]. In Brazil, there is no integrated surveillance system for recording findings identified in clinical neonatal screenings (red reflex testing, hearing screening, and screening for congenital heart diseases), and these findings are recorded individually by assistant professionals in a child's handbook [34]. To date, Brazil only has mandatory neonatal blood screening indicators [25].

The average age at which women have children has increased considerably in recent years [64, 65]. Increased maternal age has been associated with negative outcomes, such as fetal death, neonatal mortality and morbidity, and high rates of cesarean deliveries [66, 67]. Previous studies have shown that older women are more likely to have children with birth defects [68, 69]. Our results did not show a greater frequency of congenital anomalies in live births of older women, which accords with recent studies showing that, while women aged > 40 years have a greater prevalence of pregnancies with adverse events such as prematurity, miscarriage, and chromosomal syndromes, no increased prevalence of other congenital malformations was observed [28, 70, 71]. Goetzinger et al. [72] reported a significant decrease in the incidence of major fetal anomalies with increasing maternal age.

Uneducated women had higher frequencies of congenital anomalies. Health-related studies have shown that education level is a variable associated with adverse health events [73]. Low education levels have been directly related to a lower socioeconomic profile and healthcare demand, as well as reduced access to health services [74]. Low levels of maternal education, together with other related social determinants, have been associated with lower adherence to prenatal examinations and higher incidences of infectious diseases with higher adverse neonatal outcomes and a higher incidence of complications in newborns [75, 76].

To our knowledge, no studies have described the relationship between genetic factors in Indigenous women and their likelihood of developing congenital anomalies compared with non-Indigenous women. However, factors such as reduced access to health services [77, 78] and the high incidence of infectious diseases reported in Indigenous populations [78–80] are likely to have contributed to the greater number of congenital anomalies identified in newborns with Indigenous mothers in this study.

The non-inclusion of congenital anomalies among cases involving fetal deaths, which are important data, is a major limitation of this study since 3.4–20% of fetal deaths are related to congenital anomalies [81, 82]. Additionally, the actual incidences of congenital anomalies may have been underestimated due to a lack of surveillance and registration systems for congenital malformations identified during clinical neonatal screenings undertaken in the days or months after delivery [34].

Although the use of secondary data is a limitation of this study, the SINASC data from Campo Grande (the capital of Mato Grosso do Sul), which currently records approximately 40% of all births in the state [62], provides detailed and complete data documenting prematurity, type of pregnancy, type of delivery, number of prenatal consultations, and congenital anomalies [83].

One strength of our study is that the analysis was performed using an extensive database of all live births in a Brazilian state with the aim of describing the frequency, type, and factors possibly associated with the incidence of birth defects. In this cross-sectional descriptive study, we were able to compare a group of live births with no evidence of birth defects with newborns with congenital anomalies. In future, additional studies analyzing congenital anomalies separately are recommended to validate our findings.

## Conclusions

In this study, we estimated the incidence of congenital anomalies during the Zika epidemic in one region of Brazil with the highest incidences of ZIKV fever among the general population. We observed a 420% increase in the number of microcephalic cases from 2015 to 2016. Additionally, we found some associated factors that may have increased the likelihood of congenital anomalies in live-born babies of women with low or no education, Indigenous ethnicity, premature births, and twin pregnancies. Our findings can be used to support future research and the planning of intervention strategies in health facilities for better identification and medical assistance for children born with congenital anomalies.

## Supplementary Information

**Additional file 1:** **Supplementary table 1.** Frequencies of the congenital anomalies registered in SINASC according to ICD-10 categories. Mato Grosso do Sul, Brazil. 2015-2018.

**Additional file 2:** **Supplementary table 2.** Frequencies and incidences of congenital anomalies per 10,000 births according to the ICD-10 and the year of occurrence. Mato Grosso do Sul, Brazil. 2015-2018.

**Additional file 3:** **Supplementary table 3.** Frequencies of maternal, gestational, and birth data according to the ICD-10 categories of congenital anomalies. Mato Grosso do Sul, Brazil. 2015-2018.

## Data Availability

All relevant data are available within the manuscript and its additional files.
