# Supplementary material for: Congenital anomalies during the 2015–2018 Zika virus epidemic: a population-based cross-sectional study
Source: BMC Public Health. 2022 Nov 12;22:2069. doi: 10.1186/s12889-022-14490-1 (PMC9652581; doi:10.1186/s12889-022-14490-1)
Supplement: Supplementary file 1 — Additional file 1: Supplementary table 1. Frequencies of the congenital anomalies registered in SINASC according to ICD-10 categories. Mato Grosso do Sul, Brazil. 2015-2018. [file 12889_2022_14490_MOESM1_ESM.docx]

**SUPPLEMENTARY TABLE 1.** Frequencies of the congenital anomalies registered in SINASC according to ICD-10 categories. Mato Grosso do Sul, Brazil. 2015-2018.

| **Congenital anomalies** |  | | **Cumulative frequency** | | **Year** | | | | | | | |
| --- | --- | --- | --- | --- | --- | --- | --- | --- | --- | --- | --- | --- |
| **Description** | **ICD-10** | | **2015 - 2018** | | **2015** | | **2016** | | **2017** | | **2018** | |
|  |  |  | **n** | **%** | **n** | **%** | **n** | **%** | **n** | **%** | **n** | **%** |
|  |  | **1,473** | | **100** | **336** | **100** | **373** | **100** | **414** | **100** | **350** | **100** |
| Anencephaly | Q00.0 | | 37 | 2,51 | 15 | 4,46 | 8 | 2,14 | 9 | 2,17 | 5 | 1,42 |
| Encephalocele | Q01 | | 22 | 1,49 | 6 | 1,79 | 9 | 2,41 | 5 | 1,21 | 2 | 0,57 |
| Microcephaly | Q02.x | | 42 | 2,85 | 5 | 1,49 | 26 | 6,97 | 8 | 1,93 | 3 | 0,85 |
| Hydrocephaly | Q03 | | 41 | 2,78 | 11 | 3,27 | 12 | 3,22 | 13 | 3,14 | 5 | 1,42 |
| Other congenital malformations of brain | Q04 | | 15 | 1,02 | 3 | 0,89 | 4 | 1,07 | 5 | 1,21 | 3 | 0,85 |
| Spina bifida | Q05 | | 30 | 2,04 | 4 | 1,19 | 14 | 3,75 | 1 | 0,24 | 11 | 3,13 |
| Other congenital malformations of spinal cord | Q06 | | 11 | 0,75 | 2 | 0,60 | 1 | 0,27 | 7 | 1,69 | 1 | 0,28 |
| Other congenital malformations of nervous system | Q07 | | 2 | 0,14 | 2 | 0,60 | 0 | 0,00 | 0 | 0,00 | 0 | 0,00 |
| Other congenital malformations of eyelid | Q10.3 | | 6 | 0,41 | 1 | 0,30 | 3 | 0,80 | 1 | 0,24 | 1 | 0,28 |
| Microphthalmos | Q11.2 | | 5 | 0,34 | 3 | 0,89 | 0 | 0,00 | 2 | 0,48 | 0 | 0,00 |
| Congenital malformations of anterior segment of eye | Q13 | | 2 | 0,14 | 0 | 0,00 | 0 | 0,00 | 2 | 0,48 | 0 | 0,00 |
| Congenital malformations of posterior segment of eye | Q14 | | 1 | 0,07 | 0 | 0,00 | 0 | 0,00 | 1 | 0,24 | 0 | 0,00 |
| Other congenital malformations of eye | Q15 | | 15 | 1,02 | 2 | 0,60 | 3 | 0,80 | 5 | 1,21 | 5 | 1,42 |
| Congenital malformations of ear causing impairment of hearing | Q16 | | 12 | 0,81 | 2 | 0,60 | 2 | 0,54 | 4 | 0,97 | 4 | 1,14 |
| Other congenital malformations of ear | Q17 | | 65 | 4,41 | 13 | 3,87 | 11 | 2,95 | 19 | 4,59 | 22 | 6,27 |
| Other congenital malformations of face and neck | Q18 | | 25 | 1,70 | 5 | 1,49 | 6 | 1,61 | 7 | 1,69 | 7 | 1,99 |
| Congenital malformations of cardiac chambers and connections | Q20 | | 13 | 0,88 | 2 | 0,60 | 5 | 1,34 | 5 | 1,21 | 1 | 0,28 |
| Congenital malformations of cardiac septa | Q21 | | 17 | 1,15 | 3 | 0,89 | 4 | 1,07 | 9 | 2,17 | 1 | 0,28 |
| Congenital malformations of aortic and mitral valves | Q23 | | 8 | 0,54 | 2 | 0,60 | 2 | 0,80 | 4 | 0,97 | 0 | 0,00 |
| Other congenital malformations of heart | Q24 | | 38 | 2,58 | 11 | 3,27 | 8 | 2,14 | 13 | 3,14 | 6 | 1,71 |
| Congenital malformations of great arteries | Q25 | | 6 | 0,41 | 1 | 0,30 |  | 0,00 |  | 0,00 | 5 | 1,42 |
| Congenital malformations of great veins | Q26 | | 1 | 0,07 | 0 | 0,00 | 1 | 0,27 | 0 | 0,00 | 0 | 0,00 |
| Other congenital malformations of peripheral vascular system | Q27 | | 24 | 1,63 | 6 | 1,79 | 3 | 0,80 | 14 | 3,38 | 1 | 0,28 |
| Other congenital malformations of circulatory system | Q28 | | 3 | 0,20 | 1 | 0,30 | 0 | 0,00 | 1 | 0,24 | 1 | 0,28 |
| Congenital malformations of nose | Q30 | | 19 | 1,29 | 4 | 1,19 | 6 | 1,61 | 4 | 0,97 | 5 | 1,42 |
| Congenital malformations of trachea and bronchus | Q32 | | 1 | 0,07 | 0 | 0,00 | 0 | 0,00 | 0 | 0,00 | 1 | 0,28 |
| Congenital malformation of lung. unspecified | Q33.9 | | 6 | 0,41 | 1 | 0,30 | 1 | 0,27 | 2 | 0,48 | 2 | 0,57 |
| Congenital malformation of respiratory system. unspecified | Q34.9 | | 3 | 0,20 | 2 | 0,60 | 1 | 0,27 | 0 | 0,00 | 0 | 0,00 |
| Cleft palate, unspecified | Q35.9 | | 44 | 2,99 | 9 | 2,68 | 12 | 3,22 | 16 | 3,86 | 7 | 1,99 |
| Cleft lip, unilateral | Q36.9 | | 22 | 1,49 | 5 | 1,49 | 2 | 0,54 | 6 | 1,45 | 9 | 2,56 |
| Cleft palate with cleft lip | Q37 | | 30 | 2,04 | 4 | 1,19 | 9 | 2,41 | 10 | 2,42 | 7 | 1,99 |
| Other congenital malformations of tongue, mouth. and pharynx | Q38 | | 34 | 2,31 | 4 | 1,19 | 15 | 4,02 | 9 | 2,17 | 6 | 1,71 |
| Congenital malformations of esophagus | Q39 | | 15 | 1,02 | 3 | 0,89 | 3 | 0,80 | 4 | 0,97 | 5 | 1,42 |
| Other congenital malformations of upper alimentary tract | Q40 | | 3 | 0,20 | 1 | 0,30 | 1 | 0,27 | 1 | 0,24 | 0 | 0,00 |
| Congenital absence, atresia, and stenosis of large intestine | Q42 | | 32 | 2,17 | 3 | 0,89 | 19 | 5,09 | 8 | 1,93 | 2 | 0,57 |
| Other congenital malformations of intestine | Q43 | | 8 | 0,54 | 2 | 0,60 | 1 | 0,27 | 2 | 0,48 | 3 | 0,85 |
| Congenital malformations of gallbladder, bile ducts and liver | Q44 | | 3 | 0,20 | 0 | 0,00 | 0 | 0,00 | 1 | 0,24 | 2 | 0,57 |
| Other congenital malformations of female genitalia | Q52 | | 8 | 0,54 | 2 | 0,60 | 3 | 0,80 | 1 | 0,24 | 2 | 0,57 |
| Undescended and ectopic testicle | Q53 | | 9 | 0,61 | 0 | 0,00 | 3 | 0,80 | 3 | 0,72 | 3 | 0,85 |
| Hypospadias | Q54 | | 30 | 2,04 | 5 | 1,49 | 10 | 2,68 | 11 | 2,66 | 4 | 1,14 |
| Other congenital malformations of male genital organs | Q55 | | 21 | 1,43 | 6 | 1,79 | 8 | 2,14 | 5 | 1,21 | 2 | 0,57 |
| Indeterminate sex and pseudo hermaphroditism | Q56 | | 32 | 2,17 | 8 | 2,38 | 4 | 1,07 | 9 | 2,17 | 11 | 3,13 |
| Renal agenesis and other reduction defects of kidney | Q60 | | 6 | 0,41 | 0 | 0,00 | 2 | 0,54 | 2 | 0,48 | 2 | 0,57 |
| Cystic kidney disease | Q61 | | 3 | 0,20 | 1 | 0,30 | 1 | 0,27 | 0 | 0,00 | 1 | 0,28 |
| Congenital obstructive defects of renal pelvis and congenital malformations of ureter | Q62 | | 2 | 0,14 | 0 | 0,00 | 0 | 0,00 | 2 | 0,48 | 0 | 0,00 |
| Other congenital malformations of kidney | Q63 | | 4 | 0,27 | 1 | 0,30 | 1 | 0,27 | 2 | 0,48 | 0 | 0,00 |
| Other congenital malformations of urinary system | Q64 | | 2 | 0,14 | 1 | 0,30 | 0 | 0,00 | 0 | 0,00 | 1 | 0,28 |
| Congenital deformities of hip | Q65 | | 7 | 0,48 | 2 | 0,60 | 1 | 0,27 | 3 | 0,72 | 1 | 0,28 |
| Congenital deformities of feet | Q66 | | 184 | 12,49 | 49 | 14,58 | 53 | 14,21 | 37 | 8,94 | 45 | 12,82 |
| Congenital musculoskeletal deformities of head, face, spine, and chest | Q67 | | 12 | 0,81 | 1 | 0,30 | 0 | 0,00 | 4 | 0,97 | 7 | 1,99 |
| Other congenital musculoskeletal deformities | Q68 | | 19 | 1,29 | 4 | 1,19 | 6 | 1,61 | 0 | 0,00 | 9 | 2,56 |
| Polydactyly, unspecified | Q69.9 | | 120 | 8,15 | 24 | 7,14 | 30 | 8,04 | 33 | 7,97 | 33 | 9,40 |
| Syndactyly, unspecified | Q70.9 | | 15 | 1,02 | 5 | 1,49 | 2 | 0,54 | 5 | 1,21 | 3 | 0,85 |
| Reduction defects of upper limb | Q71 | | 33 | 2,24 | 7 | 2,08 | 8 | 2,14 | 7 | 1,69 | 11 | 3,13 |
| Congenital complete absence of lower limb | Q72.0 | | 14 | 0,95 | 8 | 2,38 | 1 | 0,27 | 1 | 0,24 | 4 | 1,14 |
| Other congenital malformations of limb(s) | Q74 | | 46 | 3,12 | 12 | 3,57 | 8 | 2,14 | 10 | 2,42 | 16 | 4,56 |
| Other congenital malformations of skull and face bones | Q75 | | 44 | 2,99 | 9 | 2,68 | 6 | 1,61 | 16 | 3,86 | 13 | 3,70 |
| Congenital malformations of spine and bony thorax | Q76 | | 13 | 0,88 | 0 | 0,00 | 5 | 1,34 | 2 | 0,48 | 6 | 1,71 |
| Osteochondrodysplasia with defects of growth of tubular bones and spine | Q77 | | 2 | 0,14 | 0 | 0,00 | 0 | 0,00 | 2 | 0,48 |  | 0,00 |
| Congenital diaphragmatic hernia | Q79.0 | | 9 | 0,61 | 2 | 0,60 | 1 | 0,27 | 1 | 0,24 | 5 | 1,42 |
| Exomphalos | Q79.2 | | 4 | 0,27 | 0 | 0,00 | 0 | 0,00 | 3 | 0,72 | 1 | 0,28 |
| Gastroschisis | Q79.3 | | 66 | 4,48 | 24 | 7,14 | 7 | 1,88 | 21 | 5,07 | 14 | 3,99 |
| Other congenital malformations of abdominal wall | Q79.5 | | 4 | 0,27 | 4 | 1,19 | 0 | 0,00 | 0 | 0,00 | 0 | 0,00 |
| Other congenital malformations of musculoskeletal system | Q79.8 | | 7 | 0,48 | 0 | 0,00 | 2 | 0,54 | 5 | 1,21 | 0 | 0,00 |
| Harlequin fetus | Q80.4 | | 1 | 0,07 | 0 | 0,00 | 0 | 0,00 | 0 | 0,00 | 1 | 0,28 |
| Other specified congenital malformations of integument | Q84.8 | | 9 | 0,61 | 2 | 0,60 | 0 | 0,00 |  | 0,00 | 7 | 1,99 |
| Other specified congenital malformation syndromes affecting multiple systems | Q87 | | 5 | 0,34 | 2 | 0,60 | 0 | 0,00 | 1 | 0,24 | 2 | 0,57 |
| Other congenital malformations, not elsewhere classified | Q89 | | 12 | 0,81 | 3 | 0,89 | 1 | 0,27 | 4 | 0,97 | 4 | 1,14 |
| Conjoined twins | Q89.4 | | 29 | 1,97 | 2 | 0,60 | 12 | 3,22 | 11 | 2,66 | 4 | 1,14 |
| Down syndrome, unspecified | Q90.9 | | 33 | 2,24 | 13 | 3,87 | 5 | 1,34 | 10 | 2,42 | 5 | 1,42 |
| Trisomy 18, nonmosaicism (meiotic nondisjunction) | Q91.0 | | 2 | 0,14 | 0 | 0,00 | 0 | 0,00 | 2 | 0,48 | 0 | 0,00 |
| Trisomy 13, unspecified | Q91.7 | | 1 | 0,07 | 1 | 0,30 | 0 | 0,00 |  | 0,00 | 0 | 0,00 |
| Turner's syndrome, unspecified | Q96.9 | | 3 | 0,20 | 0 | 0,00 | 0 | 0,00 | 2 | 0,48 | 0 | 0,00 |
| Chromosomal abnormality, unspecified | Q99.9 | | 1 | 0,07 | 0 | 0,00 | 0 | 0,00 | 1 | 0,24 | 0 | 0,00 |

Abbreviations: ICD-10, 10th revision of the International Classification of Diseases.

Note: Live-born babies with multiple anomalies recorded were counted once within each anomaly class.
