# Supplementary material for: Congenital anomalies during the 2015–2018 Zika virus epidemic: a population-based cross-sectional study
Source: BMC Public Health. 2022 Nov 12;22:2069. doi: 10.1186/s12889-022-14490-1 (PMC9652581; doi:10.1186/s12889-022-14490-1)
Supplement: Supplementary file 2 — Additional file 2: Supplementary table 2. Frequencies and incidences of congenital anomalies per 10,000 births according to the ICD-10 and the year of occurrence. Mato Grosso do Sul, Brazil. 2015-2018. [file 12889_2022_14490_MOESM2_ESM.docx]

**SUPPLEMENTARY TABLE 2.** Frequencies and incidences of congenital anomalies per 10,000 births according to the ICD-10 and the year of occurrence. Mato Grosso do Sul, Brazil. 2015-2018.

| **Types of anomalies** | | | | **Cases** | | | | | | | | | | **Incidence per 10.000 births.** | | | | | | | |
| --- | --- | --- | --- | --- | --- | --- | --- | --- | --- | --- | --- | --- | --- | --- | --- | --- | --- | --- | --- | --- | --- |
|  |  |  |  |  | | | | | | |  | | |  |  | | | | | | |
|  |  | **2015** | | | **2016** | | **2017** | | **2018** | | | **2015** | | | | **2016** | | **2017** | | **2018** | |
|  | **ICD-10** | | **283** | | | **282** | | **298** | | **253** | | | **63,81** | | | | **66,29** | | **66,67** | | **57,49** |
| Nervous system | Q00-Q07 | | 48 | | | 66 | | 46 | | 30 | | | 10,82 | | | | 15,44 | | 10,29 | | 6,78 |
| Eye, ear, face and neck | Q10-Q18 | | 21 | | | 18 | | 32 | | 27 | | | 4,74 | | | | 4,21 | | 7,16 | | 6,10 |
| Circulatory system | Q20-Q28 | | 20 | | | 22 | | 31 | | 14 | | | 4,51 | | | | 5,14 | | 6,94 | | 3,16 |
| Respiratory system | Q30-Q34 | | 5 | | | 0 | | 4 | | 3 | | | 1,13 | | | | 0,00 | | 0,89 | | 0,68 |
| Cleft lip and cleft palate | Q35-Q37 | | 14 | | | 22 | | 22 | | 18 | | | 3,16 | | | | 5,14 | | 4,92 | | 4,07 |
| Digestive system | Q38-Q45 | | 8 | | | 20 | | 15 | | 11 | | | 1,80 | | | | 4,67 | | 3,22 | | 2,49 |
| Genital organs | Q50-Q56 | | 13 | | | 21 | | 16 | | 12 | | | 2,93 | | | | 4,91 | | 3,58 | | 2,71 |
| Urinary system | Q60-Q64 | | 2 | | | 4 | | 1 | | 3 | | | 0,45 | | | | 0,93 | | 0,22 | | 0,68 |
| Musculoskeletal system | Q65-Q79 | | 126 | | | 98 | | 106 | | 120 | | | 28,41 | | | | 22,94 | | 23,56 | | 27,19 |
| Others | Q80-Q89 | | 12 | | | 6 | | 12 | | 9 | | | 2,71 | | | | 1,40 | | 2,68 | | 2,03 |
| Chromosomal abnormalities | Q90-Q99 | | 14 | | | 5 | | 13 | | 6 | | | 3,16 | | | | 1,17 | | 2,91 | | 1,36 |

Abbreviations: ICD-10, 10th revision of the International Classification of Diseases.
