# Supplementary material for: Congenital anomalies during the 2015–2018 Zika virus epidemic: a population-based cross-sectional study
Source: BMC Public Health. 2022 Nov 12;22:2069. doi: 10.1186/s12889-022-14490-1 (PMC9652581; doi:10.1186/s12889-022-14490-1)
Supplement: Supplementary file 3 — Additional file 3: Supplementary table 3. Frequencies of maternal, gestational, and birth data according to the ICD-10 categories of congenital anomalies. Mato Grosso do Sul, Brazil. 2015-2018. [file 12889_2022_14490_MOESM3_ESM.docx]

**SUPPLEMENTARY TABLE 3.** Frequencies of maternal, gestational, and birth data according to the ICD-10 categories of congenital anomalies. Mato Grosso do Sul, Brazil. 2015-2018.

|  | Nervous system | Eye, ear, face, and neck | Circulatory system | Respiratory system | Cleft lip and cleft palate | Digestive system | Genital organs | Urinary system | Musculoskeletal system | Chromosomal abnormalities | Other |
| --- | --- | --- | --- | --- | --- | --- | --- | --- | --- | --- | --- |
| **Age (mother)** | | | | | | | | | | | |
| 10 to 19 years old | 32  (15.9) | 19  (14,5) | 19  (17.1) | 5  (18.6) | 15  (15.6) | 19  (20.0) | 11  (11.0) | 2  (11.8) | 135  (22.6) | 7  (17.5) | 8  (14.4) |
| 20 to 34 years old | 139  (69.2) | 95  (72,5) | 65  (58.5) | 17  (62.9) | 68  (70.9) | 62  (65.3) | 79  (79.0) | 12  (70.6) | 395  (65.9) | 18  (45.0) | 37  (66.0) |
| 35 to 39 years old | 20  (9.9) | 11  (8.4) | 19  (17.1) | 4  (14.8) | 9  (9.3) | 11  (11.5) | 8  (8.0) | 2  (11.8) | 54  (9.0) | 9  (22.5) | 9  (16.0) |
| 40 years or more | 10  (5.0) | 6  (4.6) | 8  (7.3) | 1  (3.7) | 4  (4.2) | 3  (3.2) | 2  (2.0) | 1  (5.8) | 15  (2.5) | 6  (15.0) | 2  (3.6) |
| **Race/color** | | | | | | | | | | | |
| White | 54  (26.8) | 36  (27.5) | 39  (35.1) | 8  (29.6) | 30  (31.2) | 20  (21.0) | 29  (29.0) | 3  (17.7) | 151  (25.3) | 12  (30.0) | 22  (39.3) |
| No White | 124  (61.7) | 86  (65.7) | 69  (62.2) | 15  (55.6) | 58  (60.4) | 70  (73.7) | 69  (69.0) | 13  (76.5) | 417  (69.6) | 26  (65.0) | 33  (58.9) |
| Indigenous | 23  (11.5) | 9  (6.8) | 3  (2.7) | 4  (14.8) | 8  (8.4) | 5  (5.3) | 2  (2.0) | 1  (5.8) | 31  (5.1) | 2  (5.0) | 1  (1.8) |
| **Prenatal** | | | | | | | | | | | |
| No | 7  (3.5) | 5  (3.8) | 3  (2.7) | 1  (3.7) | 0  (0.0) | 0  (0.0) | 1  (1.0) | 0  (0.0) | 9  (1.5) | 0  (0.0) | 1  (1.7) |
| yes | 194  (96.5) | 126  (96.2) | 108  (97.3) | 26  (96.3) | 96  (100.0) | 95  (100.0) | 99  (99.0) | 17  (100.0) | 590  (98.5) | 40  (100.0) | 55  (98.3) |
| **Schooling** | | | | | | | | | | | |
| No | 2  (1.0) | 1  (0.8) | 4  (3.6) | 0  (0.0) | 0  (0.0) | 6  (6.3) | 3  (3.1) | 1  (5.88) | 15  (2.5) | 1  (2.5) | 2  (3.5) |
| Yes | 199  (99.0) | 130  (99.2) | 107  (96.4) | 27  (100.0) | 96  (100.0) | 89  (93.7) | 97  (97.0) | 16  (94.12) | 584  (97.5) | 39  (97.5) | 54  (96.4) |
| **Pregnancy (type)** | | | | | | | | | | | |
| Single | 186  (92.5) | 120  (91.6) | 101  (91.0) | 27  (100) | 90  (83.8) | 89  (94.7) | 96  (96.0) | 17  (100) | 580  (96.8) | 41  (100) | 49  (87.5) |
| Double or more | 15  (7.4) | 11  (8.4) | 10  (9.0) | 0  (0.0) | 6  (6.2) | 5  (5.3) | 4  (4.0) | 0  (0.0) | 19  (3.2) | 0  (0.0) | 7  (82.5) |
| **Gestational age (weeks)** | | | | | | | | | | | |
| Less than 37 | 57  (28.4) | 25  (19.1) | 19  (17.1) | 8  (29.6) | 15  (15.6) | 25  (26.3) | 23  (23.0) | 8  (47.1) | 140  (23.4) | 8  (20.0) | 19  (33.9) |
| 37 to 41) | 137  (68.2) | 101  (77.1) | 89  (80.2) | 16  (59.3) | 77  (80.2) | 66  (69.5) | 73  (73.0) | 9  (52.9) | 430  (71.8) | 30  (75.0) | 36  (64.3) |
| 42 or more | 7  (3.5) | 5  (3.8) | 3  (2.7) | 3  (11.1) | 4  (4.2) | 4  (4.2) | 4  (4.0) | 0  (0.0) | 29  (4.8) | 2  (5.0) | 1  (1.8) |
| **Gender** | | | | | | | | | | | |
| Male | 95  (49.74) | 45  (42.45) | 49  (49) | 9  (45) | 44  (50) | 41  (50.62) | 72  (82.76) | 11  (73.33) | 301  (52.99) | 17  (44.74) | 23  (46) |
| Female | 96  (50.26) | 61  (57.55) | 51  (51) | 11  (55) | 44  (50) | 40  (49.38) | 15  (17.24) | 4  (26.67) | 267  (47.01) | 21  (55.26) | 27  (54) |
| **Apgar score** | | | | | | | | | | | |
| 1º minute | | | | | | | | | | | |
| 0 to 2 | 32  (15.9) | 10  (7.63) | 14  (12.6) | 11  (40.7) | 9  (9.3) | 4  (7.4) | 5  (5.0) | 5  (29.4) | 41  (6.84) | 3  (7.5) | 9  (16.1) |
| 3 to 7 | 70  (34.8) | 32  (24.4) | 35  (31.5) | 9  (33.3) | 15  (15.6) | 16  (29.0) | 21  (21.0) | 9  (52.9) | 122  (20.4) | 8  (20.0) | 22  (39.3) |
| 8 to 10 | 99  (49.3) | 89  (67.9) | 62  (55.9) | 7  (25.9) | 72  (75.0) | 75  (63.6) | 74  (74.0) | 3  (17.6) | 436  (72.8) | 29  (72.5) | 25  (44.6) |
| 5º minute | | | | | | | | | | | |
| 0 to 2 | 31  (16.1) | 7  (6.0) | 13  (12.1) | 7  (26.9) | 11  (11.7) | 6  (6.7) | 3  (3,0) | 1  (5.9) | 28  (5.7) | 1  (2,5) | 7  (13,5) |
| 3 to 7 | 24  (12.4) | 11  (9.5) | 16  (15.0) | 7  (26.9) | 8  (8.5) | 17  (19.1) | 10  (10.0) | 3  (17.6) | 42  (8.6) | 2  (5,0) | 6  (11,5) |
| 8 to 10 | 138  (71.5) | 98  (84.5) | 78  (72.9) | 12  (46.2) | 75  (79.8) | 66  (74.2) | 87  (87.0) | 13  (76.5) | 418  (85.7) | 37  (92.5) | 39  (75.0) |
| **Birth weight (grams)** | | | | | | | | | | | |
| Less than 2.499 | 75  (37.3) | 33 (25.2) | 28  (25.2) | 6  (25.2) | 12  (12.5) | 26  (27.4) | 25  (25.0) | 4  (23.5) | 96  (16.0) | 7  (17.5) | 20  (35.7) |
| 2.500 to 3.999 | 117 (58.2) | 89 (67.9) | 77  (69.4) | 18  (69.4) | 80  (83.3) | 65  (68.4) | 70  (70.0) | 13  (76.5) | 480  (80.1) | 31  (77.5) | 33  (58.9) |
| 4.000 or more | 9  (4.5) | 9  (6.9) | 6  (5.4) | 3  (5.4) | 4  (4.2) | 4  (4.2) | 5  (5.0) | 0  (0.0) | 23  (3.8) | 2  (5.0) | 3  (5.4) |

Note: Live-born babies with multiple recorded anomalies were counted once within each anomaly class.
